# Multiplexed CRISPR/Cas9 gene knockout with simple crRNA:tracrRNA co-transfection

**DOI:** 10.1186/s13578-019-0304-0

**Published:** 2019-05-20

**Authors:** Fehad J. Khan, Garmen Yuen, Ji Luo

**Affiliations:** 1 Laboratory of Cancer Biology and Genetics, Center for Cancer Research, National Cancer Institute, Bethesda, MD USA; 2 Undergraduate Scholarship Program, National Institutes of Health, Bethesda, MD USA; 3 Master of Science in Biotechnology Program, The Johns Hopkins University Krieger School of Arts and Sciences, Washington, DC USA; 4 Present Address: Cygnal Therapeutics, Boston, MA USA

## Abstract

**Background:**

CRISPR/Cas9 mediated gene knockout is a powerful tool for genome editing with the ability to target multiple genes simultaneously. Establishing an efficient, multiplexed gene knockout system using CRISPR/Cas9 that is both simple and robust in its application would further advance the adoption of CRISPR/Cas9 for genetic studies.

**Results:**

In this study, we present a simple, versatile and highly efficient method to achieve acute gene knockout with CRISPR/Cas9 using chemically synthesized crRNA and tracrRNA oligos. We demonstrate that co-transfection of the crRNA:tracrRNA duplex into Cas9-expressing cells leads to target gene mutation and loss of target protein expression in the majority of the cell population. We also show that delivering three crRNAs targeting EGFP, KRAS and PTEN in the same reaction leads to the simultaneous knockout of all three genes. Direct comparison of multiplexed gene targeting by crRNA:tracrRNA and by siRNA indicates that these two methods are comparable in their efficiency and kinetics of gene silencing.

**Conclusions:**

Our method is a convenient yet powerful tool to enable rapid and scalable gene knockout using CRISPR/Cas9 in mammalian cells.

**Electronic supplementary material:**

The online version of this article (10.1186/s13578-019-0304-0) contains supplementary material, which is available to authorized users.

## Background

The prokaryotic clustered regularly interspaced short palindromic repeats (CRISPR)/Cas9 system is an RNA-guided endonuclease complex that utilizes short RNA guides to cleave matching viral DNA sequences in the bacterial genome [1]. In its native configuration, the Cas9 holoenzyme consists of the Cas9 endonuclease in complex with two RNAs: a 42-nucleotide (nt) CRISPR RNA (crRNA) which contains a 20-nt target-specific sequence, and a 89-nt trans-activating crRNA (tracrRNA) which serves as a scaffold for the crRNA [1]. Together, this complex recognizes an “NGG” protospacer adjacent motif (PAM) sequence 3′ to the target site that serves to anchor Cas9 binding. Strand invasion by the crRNA 20-mer and its complementation with the target DNA sequence leads to double-stranded DNA cutting by Cas9 and the subsequent mutagenesis of the target DNA [2].

CRISPR/Cas9 has been adapted for gene knockout (KO) in eukaryotic cells. In this context, it was shown that the crRNA and tracrRNA can be fused together via a short linker sequence to form a single guide RNA (sgRNA) while retaining their function [3]. This simplification enables the co-expression of sgRNA and Cas9 in host cells with either transient transfection using plasmid vectors or stable transduction using viral vectors [3]. Alternatively, CRISPR components can be delivered to host cells using transfection of a recombinant ribonucleoprotein (RNP) complex consisting of the Cas9 protein with guide RNAs [4]. Repair of Cas9-induced DNA break by the error-prone non-homologous end joining pathway often results in frame-shifting insertion and deletion (indel) mutations at the cut site that effectively knock out the expression of the target gene [3].

Compared to RNA interference (RNAi), CRISPR/Cas9-mediate gene KO has the advantage of creating true genetic nulls instead of hypomorphs. On the other hand, RNAi, particularly in the form of siRNA transfection, is easy to use due to the simplicity in its delivery. Because RNAi utilizes the endogenous RNA-induced silencing complex (RISC), gene knockdown can be achieved by simply transfecting a synthetic short-interfering RNA (siRNA) duplex into cells, a process that is both highly efficient and easily scalable. For this reason, arrayed siRNA libraries are often the method of choice for high-content screens in mammalian cells. The use of recombinant Cas9 ribonucleoprotein (RNP) transfection also avoids the need for cloning. However, the production of Cas9 RNP is considerably costlier and more cumbersome, and thus less scalable in comparison to siRNAs. Consequently, the development of arrayed CRISPR/Cas9 libraries that are analogous to arrayed siRNA libraries has significantly lagged behind. Recently, it has been shown that transfection of the RNA components in Cas9-expressing cells can lead to target gene knockout [5, 6]. These works indicate that CRISPR/Cas9 can be adapted as a transfection-based method analogous to that use for siRNAs.

Gene paralog redundancy is a common phenomenon in the mammalian genome. It is thus desirable, and necessary, to develop gene silencing platforms for co-targeting closely related, functionally redundant gene paralogs within a gene family to understand their function. In addition, a gene silencing platform that can easily co-target multiple genes simultaneously will be highly valuable at dissecting functional interactions between two or more genetic pathways to understand their interplay at the systems level. We have previously developed a siRNA-based platform that uses combinatorial siRNA pools to co-deplete up to seven genes simultaneously in human cells [7]. Currently, multiplexed gene co-targeting is a cumbersome process with CRISPR/Cas9 that either use the co-transfection of in vitro transcribed Cas9 mRNA and sgRNAs [8], co-transfection of a mixture of several Cas9 RNPs [9], co-transfection of a Cas9 vector with multiple sgRNA expressing vectors [10], and transfection or transduction using specially designed vector containing multiple sgRNA cassettes [11–14]. These approaches are less scalable and more technically demanding in their implementation compared to multiplexed siRNA transfection.

To mitigate the above practical limitations in CRISPR/Cas9, we sought to optimize a CRISPR/Cas platform that matches the efficiency and versatility of siRNA transfection. We show in this study that the transfection of a truncated crRNA:tracrRNA complex in Cas9-expressing cells can achieve very high efficiency of gene KO irrespective of the target copy number. Furthermore, we demonstrate that multiple genes can be co-ablated in the same cell using pooled crRNAs. The versatility and simplicity of our approach further diminishes the technological barrier for the adoption of CRISPR/Cas9 genome editing tools. It should also enable the development of cost-effective and high-penetrance arrayed crRNA library for high-content genetic screening.

## Results

### Efficient target gene knockout in Cas9-cells with crRNA:tracrRNA co-transfection

**Fig. 1:**
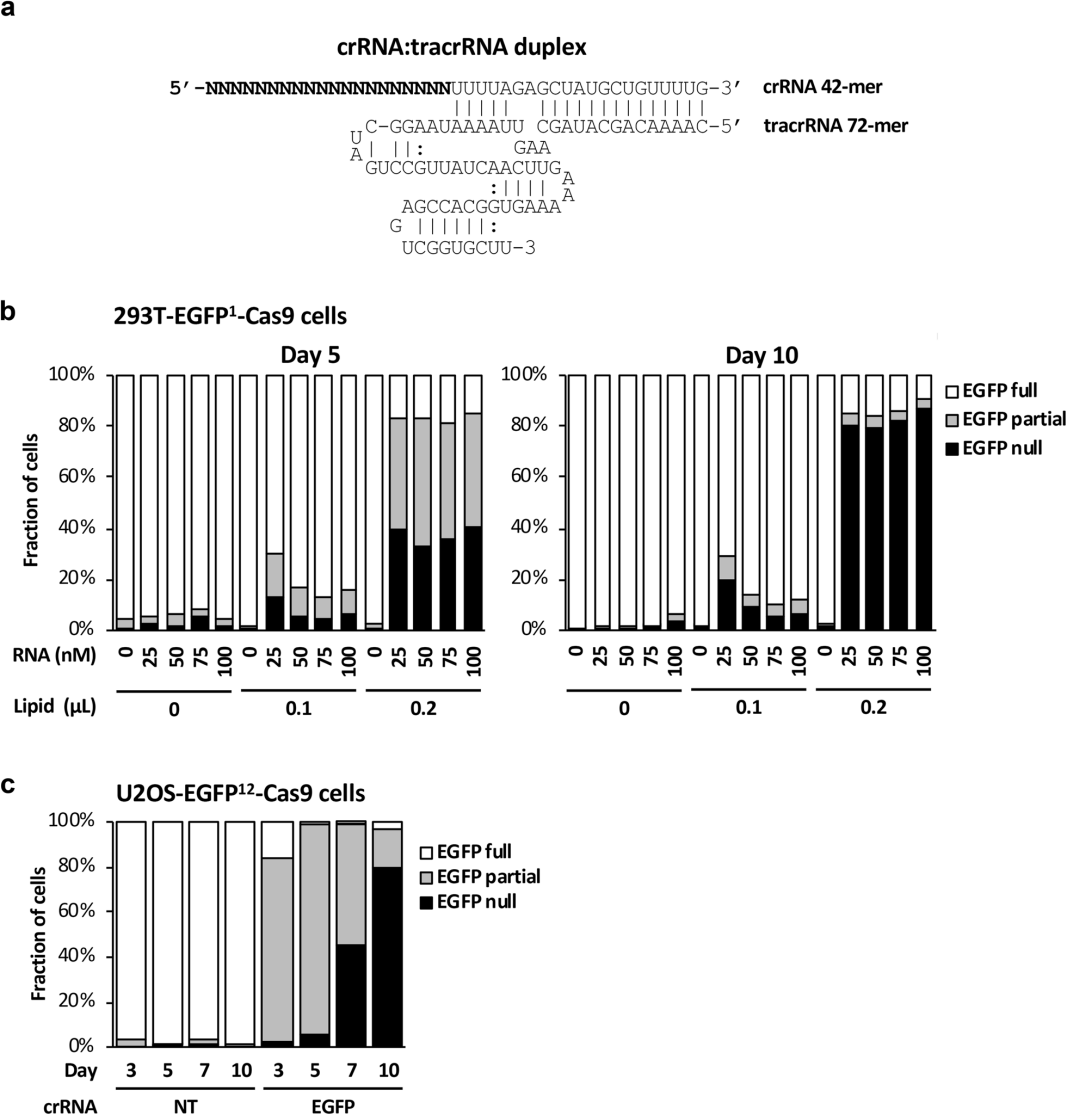
CRISPR/Cas9 mediated gene KO via RNA oligo transfection. **a** Schematics for RNA duplex design. A target-specific crRNA 42-mer and a truncated tracrRNA 72-mer were used for the transfection reaction. The target specific 20-mer sequence in the crRNA is represented by degenerate Ns. **b** Transfection of an EGFP-targeted crRNA:tracrRNA duplex in a clonal 293T-EGFP^1^-Cas9 cell line that expresses both Cas9 and a single-copy EGFP transgene. Cells in 96 well plates were reverse transfected with increasing amount of RNA and lipid as indicated. The EGFP status of cells was analyzed by flow cytometry at day 5 and day 10 post-transfection. The “EGFP full” fraction indicates cells with EGFP signal similar to untreated cells; the “EGFP null” fraction indicates cells with no EGFP signal, and the “EGFP partial” fraction indicates cells with EGFP signal that are in between the full and null gates. **c** Transfection of an EGFP-targeted crRNA:tracrRNA duplex in a clonal U2OS-EGFP^12^-Cas9 cell line that expresses both Cas9 and 12 copies of the EGFP transgene. Cells in 24 well plates were reverse transfected with 25 nM of RNA. The EGFP status of cells was analyzed by flow cytometry at indicated time points (NT, non-targeting negative control crRNA)

To develop a CRISPR/Cas9 gene KO method that is analogous to siRNA transfection, we reasoned that it should be possible to transfect the CRISPR RNA elements into cells that stably express the Cas9 apoenzyme to enable the assembly of an active Cas9 RNP. This would be analogous to the loading of a transfected siRNA into the RISC. Although sgRNA is the most common form of RNA expressed in CRISPR vectors, chemically synthesizing a 98-mer sgRNA for each guide sequence is not cost effective due to its length. We therefore used the original, two-RNA configuration and synthesized the target-specific 42-mer crRNA and the structural tracrRNA as two separate oligos. Because crystal structures of the Cas9:crRNA:tracrRNA complex indicate that 5′ region of the tracrRNA upstream the first crRNA:tracrRNA interaction and the 3′ poly-U track of the tracrRNA are neither critical for Cas9 binding nor its nuclease function [15], we utilized a truncated 72-mer version of the tracrRNA in our study to further reduce synthesis cost (Fig. 1a).

We first tested this approach in a clonal 293T-EGFP^1^-Cas9 cell line that stably expressed both Cas9 and a single copy of EGFP transgene. We used a previously validated guide RNA sequence targeting the EGFP gene [16], so we could quantify KO efficiency with flow cytometry. Next, we determined the efficiency or EGFP KO by crRNA:tracrRNA co-transfection using flow cytometry at 5 and 10 days post-transfection. We found that increasing the lipid concentration in the transfection reaction improved crRNA:tracrRNA delivery, whereas the efficiency of the system is robust over a wide range of RNA concentrations from 25 to 100 nM (Fig. 1b). Impressively, > 80% of the cells had EGFP knocked out by day 10 (Fig. 1b). Thus, co-transfection of chemically synthesized crRNA:tracrRNA is a highly effective method for acute target gene KO in bulk cell populations without further selection.

To evaluate whether the performance of this transfection-based method is sensitive to target gene copy number, we tested a clonal U2OS-EGFP^12^-Cas9 cell line that harbors 12 copies of the EGFP transgene that are randomly integrated into the genome via retroviral insertion [16]. We observed progressive loss of EGFP expression from day 3 to day 10 post-transfection by flow cytometry (Fig. 1c). Impressively, approximately 80% of the cells have completely lost EGFP expression by day 10, indicating they had all EGFP alleles deleted. Thus, transfection of chemically synthesized crRNA:tracrRNA is highly efficient at knocking out multiple copies of the target gene, consistent with what we observed with lentiviral-based stable sgRNA expression [16].

### Efficient multiplexed gene co-targeting with crRNA:tracrRNA co-transfection

**Fig. 2:**
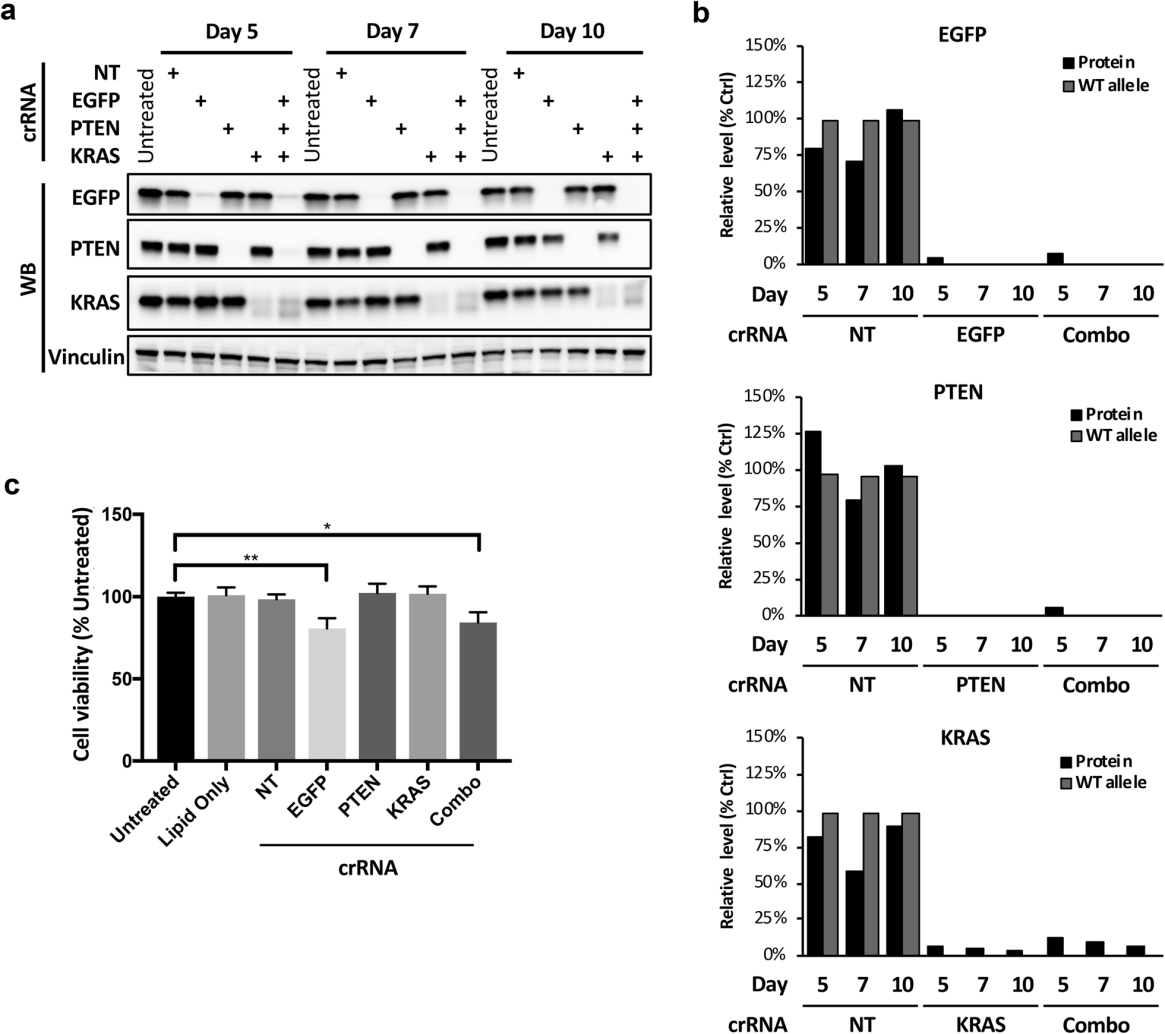
Multiplexed gene KO via crRNA combination. **a** Co-ablation of three target genes in U2OS-EGFP^12^-Cas9 cells. Cells were reverse transfected with the indicated crRNA:tracrRNA either individually or in combination. Protein expression was assessed by western at the indicated time points (Untreated, no transfection; NT, non-targeting control crRNA). **b** Quantification of target gene KO efficiency. Protein levels from **a** were quantified by densitometry. Parallel samples were collected at the indicated time points for target site indel analysis. The fraction of WT allele in the cell population was determined using TIDE (Combo, EGFP, PTEN, and KRAS crRNA combination). **c** The impact of CRISPR cutting on cell viability. Cell viability was determined 5 days after transfection using indicated crRNAs. (**p < 0.01 and *p < 0.05, two-tailed t-test)

To further examine the potency and versatility of our crRNA:tracrRNA co-transfection approach, we evaluated the capacity of this system to target multiple genes in the U2OS-EGFP^12^-Cas9 cells. In addition to crRNA targeting EGFP, we included crRNAs that target the proto-oncogene *KRAS* and tumor suppressor gene *PTEN*, both of which are diploid in this cell line. The crRNA guides for these genes were selected using a previously described sgRNA selection tool [17]. We transfected each crRNA either alone or in combination and analyzed target protein expression and target gene KO at 5, 7 and 10 days post-transfection. Western blots showed substantial loss of target protein expression as early as day 5 (Fig. 2a). Quantification of protein expression level and WT allele frequency using TIDE [18] confirmed target gene KO in most of the cells (Fig. 2b). Importantly, co-delivery of all three crRNAs were able to simultaneously knock out all three genes with very high efficiency (Fig. 2b). Thus, our data indicate that crRNAs can be combined to target multiple genes in a single transfection reaction. Previous studies have showed that multiple on-target cuts by Cas9 could reduce cell viability due to excessive DNA damage [19–21]. We therefore compared cell viability following crRNA transfection. Individual crRNAs against EGFP, PTEN and KRAS, and the combination of all three, are expected to generate 12, 2, 2 and 16 DNA breaks, respectively. We found a small but significant decrease in cell viability where multiple DNA cuts were present as indicated by the EGFP crRNA and the combination groups (Fig. 2c). Thus, higher order crRNA combinations could reduce cell viability due to increased number of DNA cuts.

**Fig. 3:**
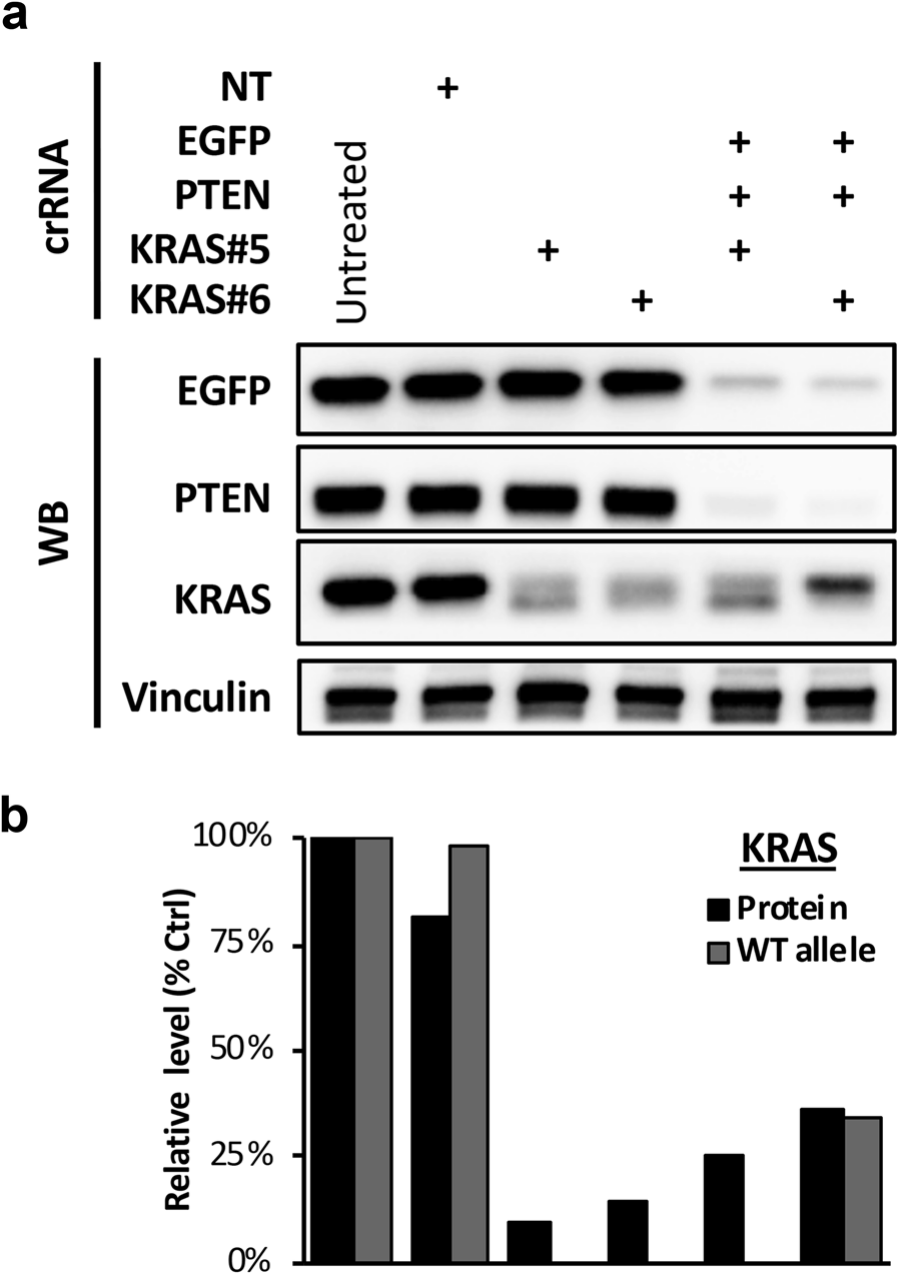
crRNA competition effect during multi-gene KO. **a** U2OS-EGFP^12^-Cas9 cells were reverse transfected with two different KRAS crRNAs (KRAS#5 and KRAS#6) either alone or in combination with EGFP and PTEN crRNAs. Protein expression was assessed by western blot 5 days post-transfection. **b** Quantification of target gene KO efficiency. Protein levels from **a** were quantified by densitometry. Parallel samples were collected on day 5 for target site mutation analysis using TIDE. The sample order in the bar chart corresponds to that of the western blot in **a**

Our previous experience with siRNA transfection indicates that potent siRNAs could interfere with each other’s activity in a combination setting [7], possibly due to a competition effect for RISC loading. We therefore evaluated whether crRNAs could also interfere with each other’s activity in a combination setting by using two distinct crRNAs against the KRAS gene (crRNA-KRAS#5 and crRNA-KRAS#6). Whereas both KRAS crRNAs were highly potent at knocking out KRAS when transfected alone, only crRNA-KRAS#5 retained its potency in the combination setting when co-transfected with EGFP and PTEN crRNAs, and the potency of crRNA-KRAS#6 was substantially reduced in the combination setting (Fig. 3). Thus, potent crRNA could potentially interfere with each other’s activity in a combination setting, and their behavior in the single and combination transfection setting need to be empirically determined.

### Comparison of performance between crRNA and siRNA mediated gene silencing

**Fig. 4:**
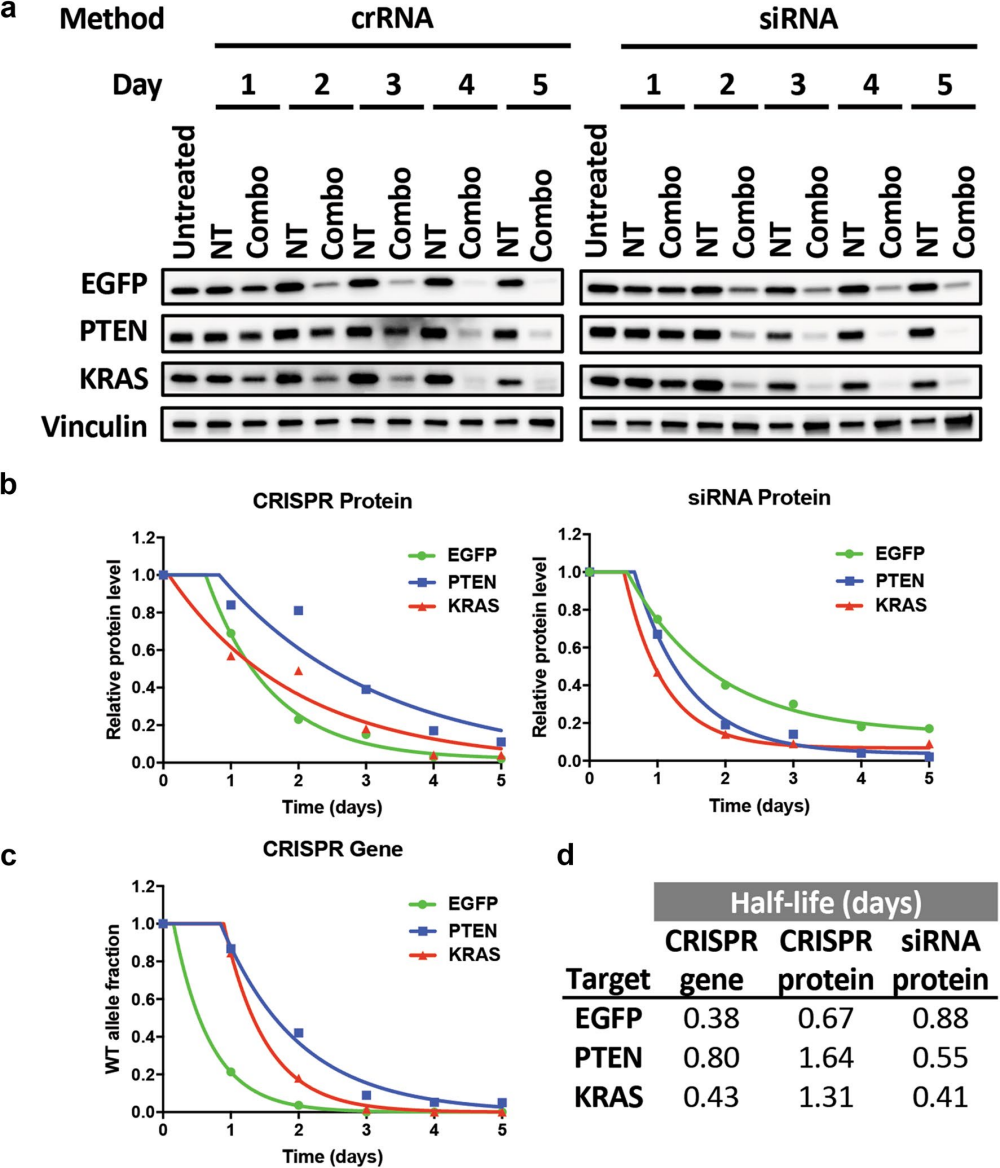
Comparison of gene silencing kinetics by crRNA and siRNA in a multiplexed setting. **a** U2OS-EGFP^12^-Cas9 cells were reverse transfected with a non-targeting control crRNA (NT), a combination of crRNAs targeting EGFP, PTEN, and KRAS (Combo), a non-targeting control siRNA pool (NT), or a combination of siRNAs targeting EGFP, PTEN, and KRAS (Combo). Protein expression was assessed by western blot at the indicated time points. **b** Quantification of protein levels from western blots in **a**. The decay of protein levels was fitted to a delayed one-phase decay model. **c** Parallel CRISPR transfection samples were collected at the indicated time points, and target site mutation was determined using TIDE. The decay of the fraction of WT allele was fitted to a delayed one-phase decay model. **d** Estimated half-lives of the EGFP, PTEN and KRAS WT allele and that of their proteins following CRISPR ablation and siRNA knockdown. Half-life was calculated using the fitted model in **b** and **c**

To better understand the kinetics of our crRNA transfection approach, we directly compared the efficiency and time course for gene silencing in U2OS-EGFP^12^-Cas9 cells using crRNAs and siRNAs target the same genes in the combination setting. Cell were transfected with crRNA:tracrRNA or siRNAs using their respective, optimized lipid transfection protocol and target protein elimination was measured during the first 5 days post-transfection. As expected, siRNAs showed rapid kinetics for protein knockdown that is noticeable by day 1 and near complete by day 2. In comparison, crRNA showed a slower kinetics with noticeable protein loss by day 2 and substantial protein loss by day 3 (Fig. 4a).

To model the kinetics of the CRISPR and siRNA reactions, we fitted the experimental data to a one-phase decay model allowing for an initial delay. The models showed that protein loss by CRISPR has a longer delay and a slower kinetics compared to that by siRNA (Fig. 4b), presumably reflecting the extra time needed for Cas9 to mutate the target DNA site in the former system and for the endogenous mRNA to decay. We also measured the reduction in WT allele frequency in the CRISPR samples and as expected, we saw mutation of the WT allele preceding the loss of proteins for all three genes (Fig. 4c). The apparent half-lives for EGFP, KRAS and PTEN WT alleles in the CRISPR samples showed similar values (Fig. 4d), indicating that Cas9 cutting and target site mutation is relatively gene-independent. The half-lives of proteins were more variable: whereas EGFP protein half-life was comparable between the CRISPR and siRNA samples, PTEN and KRAS protein half-lives were substantially longer (three to fourfold) in the CRISPR samples than in the siRNA samples. A possible explanation is that their mRNAs may have different decay rates.

## Discussion

In this study, we sought to simplify CRISPR/Cas9 mediated gene KO to obviate the need for vector construction. Our motivation was to develop a simple, cost-effective, efficient and scalable gene KO approach that parallels the simplicity of siRNA transfection. We showed that the co-transfection of a target-specific crRNA 42-mer and a truncated structural tracrRNA 72-mer is highly efficient at knocking out target genes, either singularly or in combination, in bulk cell populations within 3 days. Our current study, together with recent works demonstrating success in knocking out cell cycle genes with similar approaches [5, 6], firmly establishes crRNA:tracrRNA transfection as a simply yet powerful method to implement CRISPR/Cas9 for gene knockout studies. Below we discuss the potential utility and limitations of this approach.

**Fig. 5:**
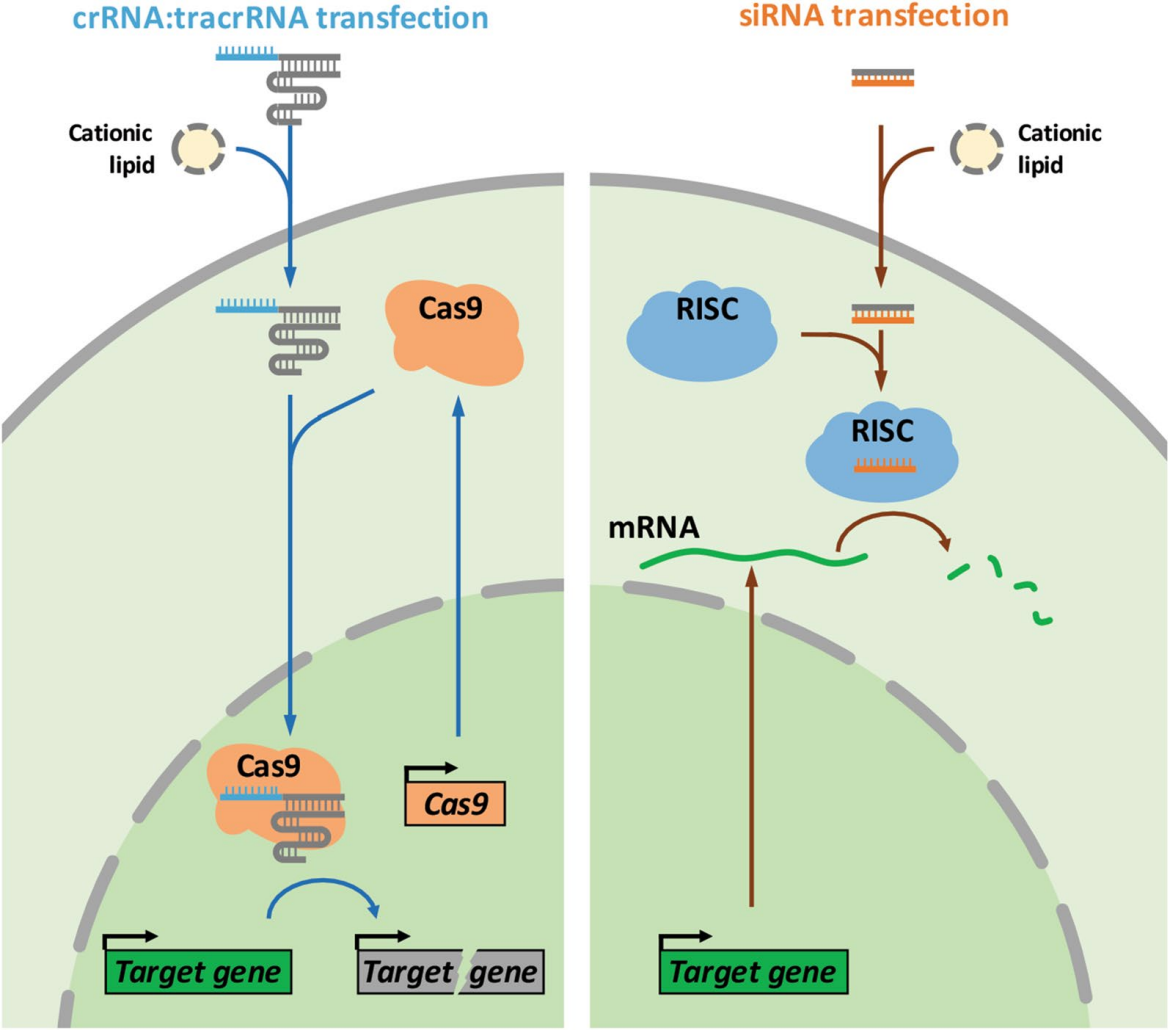
Schematics of crRNA transfection-mediated gene KO. Our crRNA:tracrRNA transfection method parallels that for siRNA transfection. The RNA components for CRISPR are delivered to Cas9-expressing cells using cationic lipid transfection. They assemble with Cas9 protein to form the active Cas9 RNP which in turn directs target gene cleavage

The most salient advantage of RNA transfection is that it dramatically simplifies the use of CRISPR/Cas9 for gene KO studies. A pre-requisite for an efficient CRISPR/Cas9 systems appears to be the establishment of clonal cell lines that stably express Cas9 and show efficient sgRNA-directed cutting at the target site. We typically identify such clones through empirical testing of multiple clones following stable lentiviral Cas9 transduction. Once such Cas9-positive cell lines are established, they can be transfected with crRNA:tracrRNA duplex to achieve highly efficient gene KO. At the mechanistic level, we speculate that newly synthesized Cas9 apoprotein can complex with the transfected crRNA:tracrRNA in the cytoplasm to form active Cas9 RNPs, which subsequently translocate into the nucleus to elicit target mutation (Fig. 5). We showed that this system performed with consistent efficiency at knocking out multiple copies of a stably integrated EGFP transgene and two endogenous genes KRAS and PTEN. This indicates that a single round of transfection can deliver sufficient RNA and a generate sufficient number of active Cas9 RNPs to mutate all target sites without needing to replenish the RNA oligos. Compared to transfection using recombinant RNPs, our approach requires only chemically synthesized short RNA oligos, thus reducing both the cost and batch-to-batch variations associated with reagent generation.

RNA transfection shows both high efficiency and rapid kinetics and these properties that are highly desired for acute gene KO studies. Because the size of the crRNA and tracrRNA is comparable to that of siRNAs, they can be transfected into cells at high efficiency with conventional cationic lipids. Our data indicate that the majority of cells can be transfected. Importantly, in transfected cells gene deletion tend to be mostly homozygous, even when target copy number was high, as demonstrated by the successful knockout of EGFP in the U2OS-EGFP^12^-Cas9 cells. This enables the analysis of cellular phenotype in bulk populations without the need for further selection or clonal derivation. Our time course analysis of three targets (EGFP, KRAS, and PTEN) showed that target gene KO and protein elimination occurs within 3 days post-transfection, even for a relatively long half-life protein such as KRAS. This represents a time delay of only 1 day in comparison to siRNA mediated gene silencing. Thus, crRNA:tracrRNA transfection enables the assessment of acute phenotypes within a time frame of 5–6 days—a time frame that is comparable to typical siRNA experiments.

The crRNA:tracrRNA transfection method is easily scalable. Because sequence-specific crRNAs can be chemically synthesized in an arrayed format and the tracrRNA can be synthesized in bulk, this platform can be easily scaled to create genome-wide, arrayed CRISPR libraries that are analogous to arrayed siRNA libraries for high-content screens. Indeed, proof-of-concept screens of this type have been carried out to discover cell cycle regulator genes [5]. This approach, therefore, should enable the rapid construction and deployment of arrayed CRISPR libraries to complement pooled lentiviral CRISPR libraries for genetic screens.

Importantly, we demonstrated that crRNAs can be multiplexed in a transfection reaction. This feature has several important utilities. For example, crRNAs can be combined to target one or more closely related gene orthologs to overcome their functional redundancy and dissect gene family function. Furthermore, crRNAs targeting genes in different pathways can be combined to interrogate the functional interaction between two or more pathways, thus enabling the study of network topology at the systems level. We have previously demonstrated the utility of multiplexed gene targeting using siRNA combinations [7]. This study demonstrated that multiplexed gene targeting can also be achieved using crRNA combinations. In the context of arrayed CRISPR library construction, combining two or three sgRNAs that target the same gene in one pool could potentially increase the success of gene KO and improve the penetrance of the library.

We note that multiplexing crRNAs could lead to DNA damage-induced cytotoxicity in cells as it has been described previously [19–21]. Thus, the complexity of higher order crRNA combinations might be ultimately limited by the number of double-stranded DNA breaks a cell line could tolerate and combinatorial crRNA knockdown may only be feasible in cell lines that are efficient at repairing DNA breaks. We also noted that, when crRNAs were co-transfected together, there could potentially be a competition effect where a crRNA that is potent on its own became less potent in a combination with other potent crRNAs. This is analogous to what we observed with siRNA competition in higher order combinations [7]. Thus, for crRNAs to be effectively utilized in a combination setting, their KO efficiency should be empirically determined in a combination setting rather than in an individual setting. How crRNAs interfere with each other’s activity is currently unclear, a plausible mechanism is that they compete for a limited availability of Cas9 apoenzyme. Such competition for Cas9 loading could be tested using RIP-seq in a future study.

In conclusion, we have implemented an RNA transfection-based CRISPR platform that parallels the simplicity, efficiency, cost-effectiveness, scalability and multiplexity of siRNA transfections. This method should further reduce the technological barrier for the implementation of CRISPR/Cas9 in genetic studies. We envision that the versatility of this transfection-based platform could be further enhanced by including a DNA template for homologous recombination in the transfection mixture to enable precision gene editing. This would provide a powerful means to rapidly create large panels of isogenic cell lines with complex genotypes for functional study. It is also worth noting that crRNA:tracrRNA transfection should work with both CRISPRi and CRISPRa technologies since they share the same targeting principle. Further development of this platform in these directions could lead to additional CRISPR/Cas9 tools for gene manipulation.

## Methods

### Cell culture

HEK 293T cells were cultured in Dulbecco’s Modified Eagle’s Medium (Lonza #12-604Q), and U2OS cells were cultured in McCoy’s 5A Medium (Lonza #12-688F). Both media were supplemented with 10% heat-inactivated fetal bovine serum (Gibco #10438026) and 1% penicillin/streptomycin (Lonza 17-602E) and cultured at 37 °C in humidified 5% CO_2_ incubator. The 293T-EGFP^1^ and U2OS-EGFP^12^ cell lines that express one and 12 copies of retrovirally integrated EGFP transgene were previously described [16]. Cas9 was introduced into these cells by lentiviral transduction using a LentiCas9-Blast vector (Addgene Plasmid #52962) packaged with standard protocols [16]. Transduced cells were selected with blasticidin (10 μg/mL), and single cells were sorted into a 96-well plate by flow cytometry. Clonal cell lines were expanded and Cas9 protein expression level was determined by western blot. Clones with high Cas9 expression were used for subsequent experiments.

### Oligonucleotide and antibody information

A list of crRNA, tracrRNA, siRNAs, PCR primers and antibodies used in this study are listed in Additional file 1. The crRNA targeting EGFP has been described previously [16]. For crRNAs targeting PTEN and KRAS, they were selected using the Broad Institute online sgRNA design tool (https://portals.broadinstitute.org/gpp/public/analysis-tools/sgrna-design) [17].

### RNA transfection and cell viability assay

Each RNA oligos suspended in RNAase-free water at a stock concentration of 100 μM. Reverse transfection was used to deliver crRNA and tracrRNA into cells. Equimolar amounts of crRNA and tracrRNA were used to achieve the desired final concentration in the reaction, which was 25 nM for all experiments except the initial optimization studies. The crRNA:tracrRNA mixture was first stamped into wells and spun down briefly at 2000 RPM in a swing-arm centrifuge (Beckman Coulter Allegra X-12R Centrifuge) at room temperature. We tested several lipid transfection reagents and found that Lipofectamine MessengerMAX (ThermoFisher Scientific #LMRNA008) and Lipofectamine RNAiMAX (ThermoFisher Scientific #13778150) were the most effective in our experiments. The respective lipids were incubated in serum-free Opti-MEM media (Gibco #31985070) and the RNA stamped plates for the length of time outlined in the manufacture’s protocol. Trypsinized cells suspension were diluted in medium supplemented with 15% heat inactivated fetal bovine serum and 1% penicillin/streptomycin and added to the wells. The volume of cells to lipid/RNA mixture was determined such that the final concentration of serum in the media was 10%. Cells were spun down at 800 RPM for 5 min and cultured for the desired period of time.

Reverse transfection of 293T-EGFP^1^-Cas9 cells was carried out in 96-well plates where 5 μL of crRNA:tracrRNA duplex (0–100 nM), 50 μL of serum-free media with lipid (0–0.2 μl), and 800 cells suspended in 105 μL of Dulbecco’s Modified Eagle’s Medium (Lonza #12-604Q) to a total volume of 160 μL per well. It was determined that 25 nM of crRNA:tracrRNA duplex and 0.2 μL lipid was optimal for these cells. Subsequent reverse transfection studies in U2OS cells were performed in 24-well plates and reagents were scaled accordingly. Each well contained 25 nM of oligonucleotide in 30 μL of water, 1.2 μL of lipid in 300 μL of Opti-MEM, and 6000 U2OS cells suspended in 630 μL of McCoy’s 5A media to a total volume of 960 μL per well. For the crRNA combination studies, 25 nM of each crRNA and 75 nM of tracrRNA was used. A non-targeting crRNA was used a negative control. The crRNA siRNA comparison reverse transfection was performed using 30,000 U2OS cells per well in 24-well plates. Reverse transfection of siRNA was performed with 10 nM siRNA per gene per well, and Lipofectamine RNAiMAX (ThermoFisher Scientific #13778150) was used at 1.2 μL per well. Non-targeting siRNAs (siNeg, Qiagen # 1027281) was used a negative control. For longer experiments, cells were passaged and expanded at day 5 post-transfection into 6-well plates.

Cell viability was determined by using the ATP-based CellTiter-Glo Luminescent Cell Viability Assay (Promega #G8462) at day 5 post-transfection following the manufacture’s protocol.

### Flow cytometry, Western blot, and target site indel analysis

Flow cytometry was performed as described previously [16]. Briefly, cells were trypsinized at the desired time points and their EGFP signals were quantified on a flow cytometer (BD Biosciences FACS Calibur). The EGFP null gate was set using parental cells lines without EGFP expression and thus identifies cells that have lost EGFP expression completely. The EGFP full gate was set using EGFP-expressing cells without any treatment. The EGFP partial gate defines cells whose EGFP signal is between the null and full gates. All analysis was performed using FlowJo.

For protein expression analysis, cells were lysed with 1X Laemmli sample buffer (Bio-Rad #1610747) at the desired time points. Lysates were then denatured at 95 °C for 5 min. Samples were run on 8–16% SDS-PAGE gels (BioRad Cat # 5671105) and transferred to a nitrocellulose membrane for immunoblotting. Membranes were probed with primary antibodies (Additional file 1) and HRP-conjugated secondary antibodies. Band densitometry was determined using a ChemiDoc MP Imaging System (BioRad) with its associated Image Lab software. Protein level in each sample was first adjusted using its respective loading control and then normalized to the negative control sample.

Genomic DNA was extracted from cells collected at the desired time points using the DNeasy Blood and Tissue Kit (Qiagen #69504). PCR was performed with the CloneAmp HiFi PCR Premix (Takara #639298) using 30 ng of genomic DNA and target specific primers for each gene (Additional file 1). Gel electrophoresis of the PCR amplicon was gel purified using the QIAquick Gel Extraction Kit (Qiagen #28706) and analyzed by Sanger sequencing. Chromatograms were analyzed using the TIDE program (https://tide.nki.nl/) to determine indel frequency.

### Data modeling

Prism 8 was used to perform unpaired two-tailed tests and model the decline rate of protein and WT alleles. A delay followed by one phase decay model was used for all analysis. To best fit the data, the initial delay was constrained to be < 1 day for the CRISPR protein and allelic data and < 0.5 day for the siRNA protein data.

## Additional file

**Additional file 1: Table S1.** CRISPR RNA oligo sequences used in this study. The tracrRNA was synthesized by IDT as a Ultramer RNA Oligo. All crRNAs were synthesized by Sigma Aldrich with HPLC purification. **Table S2.** siRNA sequences used in this study. **Table S3.** PCR primer sequences. These primers were used for PCR amplification of the CRISPR/Cas9 target site in each gene. **Table S4.** Antibodies information.

## Data Availability

Cell lines and other reagents described in this study will be made available upon request.

## Abbreviations

CRISPR: clustered regularly interspaced short palindromic repeats
crRNA: CRISPR RNA
EGFP: enhanced green fluorescent protein
indel: insertion and deletion
KO: knockout
NT: non-targeting
nt: nucleotide
PAM: protospacer adjacent motif
RISC: RNA-induced silencing complex
RNAi: RNA interference
RNP: ribonucleoprotein
sgRNA: single guide RNA
siRNA: short-interfering RNA
tracrRNA: trans-activating crRNA
TIDE: Tracking Indels by DEcomposition
WT: wild-type
