# Supplementary material for: Multiplexed CRISPR/Cas9 gene knockout with simple crRNA:tracrRNA co-transfection
Source: Cell Biosci. 2019 May 20;9:41. doi: 10.1186/s13578-019-0304-0 (PMC6528186; doi:10.1186/s13578-019-0304-0)
Supplement: Supplementary file 1 — Additional file 1: Table S1. CRISPR RNA oligo sequences used in this study. The tracrRNA was synthesized by IDT as a Ultramer RNA Oligo. All crRNAs were synthesized by Sigma Aldrich with HPLC purification. Table S2. siRNA sequences used in this study. Table S3. PCR primer sequences. These primers were used for PCR amplification of the CRISPR/Cas9 target site in each gene. Table S4. Antibodies information. [file 13578_2019_304_MOESM1_ESM.pdf]

Additional file 1

Table S1. CRISPR RNA oligo sequences used in this study. The tracrRNA was synthesized by IDT as a Ultramer RNA Oligo. All crRNAs were synthesized by Sigma Aldrich with HPLC purification.

Description	Target	Sequence (5'-3')
tracrRNA	n/a	CAAAACAGCAUAGCAAGUUAAAAUAAGGCUAGUCCGUUAUCAACUUG AAAAAGUGGCACCGAGUCGGUGCUU
crRNA-NT	none	CCAUAUCGGGGCGAGACAUGGUUUUAGAGCUAUGCUGUUUUG
crRNA-EGFP	EGFP	GGGCGAGGAGCUGUUCACCGGUUUUAGAGCUAUGCUGUUUUG
crRNA-PTEN	Human *PTEN*	UGUGCAUAUUUAUUACAUCGGUUUUAGAGCUAUGCUGUUUUG
crRNA-KRAS#5	Human *KRAS*	AGAGGAGUACAGUGCAAUGAGUUUUAGAGCUAUGCUGUUUUG
crRNA-KRAS#6	Human *KRAS*	UCUCGACACAGCAGGUCAAGGUUUUAGAGCUAUGCUGUUUUG

Table S2. siRNA sequences used in this study.

Description	Target	Manufacturer (Cat #)	Sense Stand Sequence
siNeg	n/a	Qiagen (1027281)	n/a (pool)
siEGFP	EGFP	Invitrogen (1022064)	CGGCAAGCTGACCCTGAAGTTCAT
siPTEN	Human *PTEN*	Dharmacon (D-003023-05)	GUGAAGAUCUUGACCAA
siKRAS	Human *KRAS*	n/a	UUGACGAUACAGCUAAUUCAUA

Table S3. PCR primer sequences. These primers were used for PCR amplification of the CRISPR/Cas9 target site in each gene.

Description	Sequence (5'-3')
EGFP Forward	AAGCCCTTTGTACACCCTAAGCCTCC
EGFP Reverse	GCTTGTCGGCCATGATATAGACGTTGTG
PTEN Forward	CTCTGGAATCCAGTGTTTC
PTEN Reverse	GATCTTAAATGCTTTCACCCTGG
KRAS Forward	GCCATTTGTCCGTCATCTTTG
KRAS Reverse	GATGCAGTCTGGAGCAAGTTA

Table S4. Antibodies information.

Protein	Manufacturer (Cat #)	Dilution
EGFP	Santa Cruz (sc-8334)	1/500
PTEN	Cell Signaling (9188)	1/1,000
KRAS	Sigma Aldrich (WH0003845)	1/200
Vinculin	Sigma Aldrich (V9131)	1/10,000
